# Individual variation in unfractionated heparin dosing after pediatric cardiac surgery

**DOI:** 10.1038/s41598-020-76547-8

**Published:** 2020-11-10

**Authors:** Keiko Hikino, Masaru Koido, Kentaro Ide, Nao Nishimura, Chikashi Terao, Taisei Mushiroda, Satoshi Nakagawa

**Affiliations:** 1 Division of Critical Care Medicine, Department of Critical Care and Anesthesia, National Center for Child Health and Development, 2-10-1 Okura, Setagaya-ku, Tokyo, 157-8535 Japan; 2 Laboratory for Pharmacogenomics, RIKEN Center for Integrative Medical Sciences, 1-7-22 Suehiro-cho, Tsurumi-ku, Yokohama City, Kanagawa 230-0045 Japan; 3 Laboratory for Statistical and Translational Genetics, RIKEN Center for Integrative Medical Sciences, 1-7-22 Suehiro-cho, Tsurumi-ku, Yokohama City, Kanagawa 230-0045 Japan

**Keywords:** Medical research, Paediatric research

## Abstract

We aimed to identify attributing factors to the interindividual variabilities of the infusion rates in unfractionated heparin therapy. We included patients who required unfractionated heparin therapy to achieve the target APTT after cardiac surgery between May 2014 and February 2018. Fifty-nine patients were included, of whom 8 underwent Blalock-Taussig shunt; 27, Glenn procedure; 19, Fontan procedure; 3, mechanical valve replacement; and 2, Rastelli procedure. Previously reported variables that influenced the response to unfractionated heparin treatment were initially compared, which included age; weight; sex; type of surgery; platelet count; fibrinogen, antithrombin III, total protein, albumin, alanine transaminase, and creatinine levels; and use of fresh frozen plasma. The type of surgical procedure was found to be significantly associated with the differences in heparin infusion rate (P = 0.00073). Subsequently, the variance explained by these factors was estimated through a selection based on the minimum Akaike information criterion value; models constructed by various combinations of the surgery types were compared. The model including the Blalock-Taussig shunt, Glenn procedure, and mechanical valve replacement showed the highest summed variance explained (29.1%). More than 70% of the interindividual variability in initial heparin maintenance dosing was unexplained.

## Introduction

Unfractionated heparin therapy is commonly administered after cardiac surgery for anticoagulation in intensive care units. Typically, each institution has its own protocol for providing optimal unfractionated heparin therapy to achieve the target activated partial thromboplastin time (APTT) or activated clotting time (ACT) when patients are receiving extracorporeal membrane oxygenation (ECMO). Although studies have shown that unfractionated heparin therapy is more strongly associated with anti-Xa activity, measurement of anti-Xa activity as a routine practice is not necessarily the standard of care due to lack of evidence^1–4^.

Intensivists frequently have difficulty predicting responses to unfractionated heparin therapy in each patient by observing contributing factors to the interindividual variabilities of the unfractionated heparin dosing required for each patient. These interindividual variabilities are well known, and previous studies reported multiple factors, including obesity, aging, hepatic or renal disease, altered production of heparin-binding proteins, general heparin resistance, antithrombin deficiency, increased heparin clearance, elevated levels of heparin-binding proteins, and increased plasma levels of factor VIII, fibrinogen, and platelet factor 4^5–11^. Recently, several studies have been conducted to explain interindividual variabilities of unfractionated heparin therapy, one of which is by Al-Sallami et al. who developed a population pharmacokinetics (PK)-pharmacodynamics (PD) model in pediatric patients during cardiac angiography and showed that fat-free mass was a significant covariate for clearance^12^. The study by Delavenne et al. developed a population PK-PD model for adults patients during cardiopulmonary bypass (CPB) and reported that the inclusion of body weight in their model decreased the interindividual variabilities of clearance and central compartment volume^13^. However, existing unfractionated heparin dosing algorithms do not incorporate these factors.

Thus, in this study, we aimed to identify the attributing factors to the interindividual variabilities of the heparin infusion rate upon achieving the target APTT, which is one of the most important phenotypes in the intensive care unit and can also be used to quantify responses to unfractionated heparin therapy.

## Methods

We aimed to identify the attributing factors to the interindividual variabilities of the heparin infusion rate upon achieving the target APTT. We performed a retrospective observational cohort study to examine the medical records of patients admitted to the pediatric intensive care unit (PICU) in a single tertiary care center (National Center for Child Health and Development [NCCHD], Tokyo, Japan) between May 2014 and February 2018. This study was approved by the ethics review board of the National Center for Child Health and Development, Tokyo, Japan (Receipt No. 2248). Written informed consent was waived by National Center for Child Health and Development [NCCHD], Tokyo, Japan because of the retrospective design. All methods were carried out in accordance with relevant guidelines and regulations. The inclusion criteria of this study were as follows: (1) patients who were receiving unfractionated heparin therapy; (2) patients admitted to the PICU after cardiac surgery (Blalock-Taussig shunt, Glenn procedure, Fontan procedure, biological or mechanical valve replacement, or Rastelli procedure, as we provide postoperative heparin therapy with target APTT ranges of 40 to 65 s, depending on the surgical procedure as shown in Supplementary Table 1, only for patients with postsurgical status); and (3) patients who required titration of the heparin infusion rate to achieve the target APTTs. We considered that the target therapeutic ranges of APTT had been reached when the APTT was within ± 5 s of the target APTT ranges in the Supplementary Table 1. We excluded patients who receiving ECMO or any dialysis. In addition, we excluded patients who did not reach the target APTT after 72 h of starting heparin therapy, as we usually transition to either warfarin or aspirin from unfractionated heparin for anticoagulation therapy once enteral feeding is successfully started. If patients had multiple admissions during the study period, we used the medical records from the first admission or those with full descriptions of the postoperative course in the progress notes.

### Drug administration

In the PICU, pediatric intensivists started unfractionated heparin therapy at an initial rate of 10 units/(kg h). They titrated the unfractionated heparin dosage to achieve the target APTT ranges. All the doses were recorded, including the start time of the infusions.

### Definitions of outcomes

Given the fact that a steady-state drug concentration in the blood is, in general, achieved after 4 or 5 half-lives of drug elimination, for unfractionated heparin with a half-life of 0.5–2 h, the steady state would be reached 2–10 h after treatment initiation^14^. More importantly, it is well known that APTT reaches a steady state after approximately 4 h in children^10^. We excluded patients who did not reach the target APTT after 72 h of starting heparin therapy as mentioned above. Therefore, we set the primary outcome for our study to be the initial maintenance dosage of unfractionated heparin (unit/[kg h]) given to a patient between 4–72 h of starting heparin therapy. In addition, we considered the infusion rate of heparin maintenance dosage, summing all the infusion rates of heparin running in lines such as arterial lines or central venous catheters to measure blood pressure, including systemic blood and pulmonary artery pressures. In the PICU, unfractionated heparin was administered via an arterial line at a rate of 2 units/h for patients whose body weights were < 5 kg, via an arterial line at 4 units/h for those whose body weights were ≥ 5 kg, and via a central venous catheter at a rate of 2 units/h for all the patients to prevent clotting. In addition, given that the initial APTT could be largely influenced by heparin therapy during surgery, we considered the target APTT as follows: the APTT (1) reached nadir after admission to the PICU and (2) subsequently reached the target.

### Data collection

The baseline demographic data included age (years), weight (kg), height (cm), sex, name of performed surgical procedures, and name of cardiac diseases for which those surgical procedures were collected. Laboratory data, concomitant drugs administered, number of transfusions given, and numbers/types of lines used, such as arterial lines or central venous catheter information, were also collected.

### Statistical analyses

**Figure 1:**
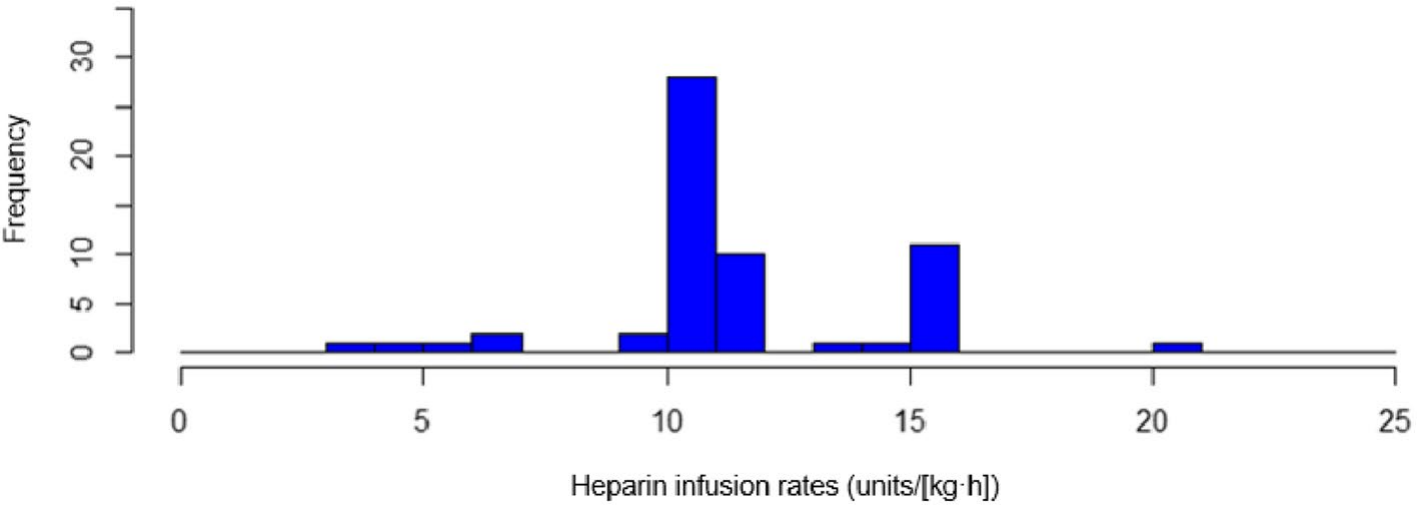
Histogram of heparin infusion rates (units/[kg h]) in all the patients upon reaching their target APTTs. The Shapiro-Wilkes test results show an abnormal distribution (P = 3.1E−06).

The Shapiro-Wilkes test was used to evaluate the normality of the heparin infusion rate. Given that the histogram of the heparin infusion rates showed an abnormal distribution (P = 3.1E−06; Fig. 1), we log-transformed the heparin infusion rate for further analysis. For correlation analysis, the Spearman *ρ* value was used.

As covariates of the interindividual variabilities of the infusion rate of heparin as maintenance therapy after cardiac surgery, the factors that were previously reported to be associated with heparin response/resistance and for which data are currently available were included in our study. We set the cutoff antithrombin III activity level to ≤ 60%, platelet count to > 300,000, and increased factor VIII and fibrinogen levels^5–9^. We set the cutoff the fibrinogen level to > 300 mg/dL in this study^15^. In addition, we investigated body weight, types of surgical procedure, total protein level (> 5.0 or < 5.0 g/dL), albumin level (> or < 3.0 g/dL), alanine transaminase level (> 100 or < 100 IU/L) to represent liver function; serum creatinine level (> 0.8 or < 0.8 mg/dL) to represent renal function, use of fresh frozen plasma (but limited to occasions within 48 h before reaching the target APTT), use of platelet transfusion (only within 48 h before the target APTT), and use of protamine (within 48 h before the target).

Subsequently, we selected covariates with significant associations that were determined by conducting nonparametric tests, specifically the Wilcoxon rank-sum test for differences between two groups and the Kruskal–Wallis test for comparing three or more variables. We then constructed a polynomial linear regression model and evaluated the total explained variance by using the variables with significant associations in the covariate selection to investigate how much interindividual variabilities of unfractionated heparin therapy could be explained by those covariates. When the types of surgical procedure to be accounted for in the model development are required, we used a dummy variable for the Rastelli procedure.

In addition, the Akaike information criterion (AIC) for each model was calculated in order to identify the optimal model in terms of possible prediction ability^16^. We selected only the polynomial regression models where the directions of the regression coefficients and values that multiply the predictor values were estimated to be the same as the direction of the regression coefficient in the single regression model for each variable.

We also estimated the variance explained, adjusting for the APTT in each model and regressing the effect of APTT, as APTT could also be influenced by multiple factors and variabilities could be due to APTT itself^7^. All statistical analyses were two-sided, and a P value of < 0.05 was considered significant. All analyses were performed using the R version 3.5.0 statistical software^17^. Boxplots were drawn using the R package ggplot2^18^.

Given that the target APTT is slightly higher after mechanical valve replacement, as shown in Supplementary Table 1, we conducted additional subgroup analyses, excluding patients with mechanical valve replacement.

### Ethics approval and consent to participate

This study was approved by the ethics review board of the National Center for Child Health and Development (Receipt No. 2248).

## Results

**Figure 2:**
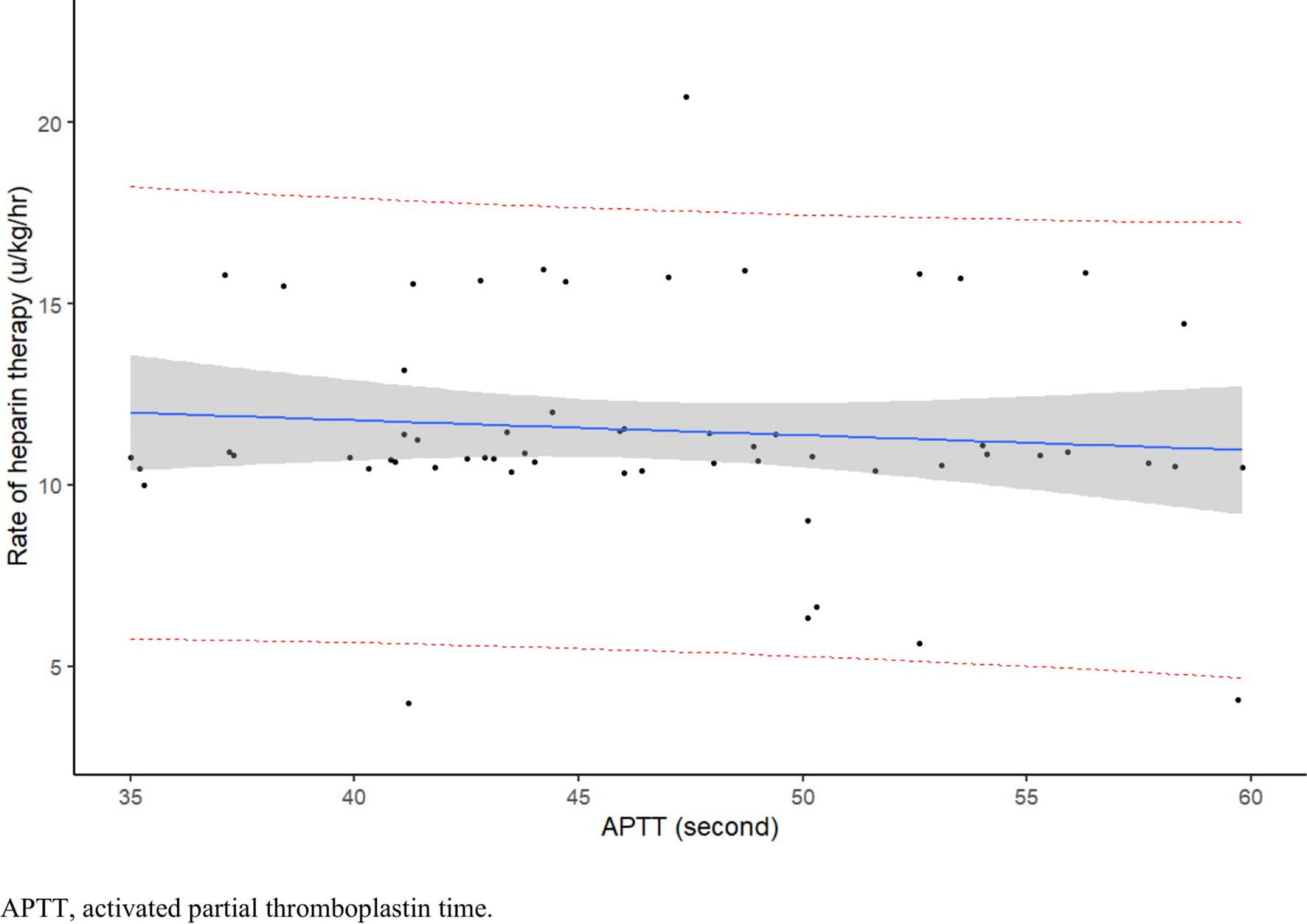
Heparin infusion rates upon achieving the target APTTs for the 59 patients. Each dot represents 1 patient. The blue line is a regression line. The gray area represents 95% confidence intervals. The red dotted line represents 95% prediction intervals.

Ninety-two unique patients received heparin therapy after cardiac surgery (Blalock-Taussig shunt, Glenn procedure, Fontan procedure, biological or mechanical valve replacement, or Rastelli procedure, as we provide postoperative heparin therapy with target APTTs only for patients with postsurgical status) to achieve the target APTTs at the PICU in the NCCHD between May 2014 and February 2018. Among the patients, 30 were excluded because their target APTTs were not reached within 72 h of starting heparin therapy. In addition, we excluded 3 patients whose ages were considered to be outliers (17, 20, and 22 years) as compared with the ages of the other patients that ranged from 0 to 4 years. Thus, 59 unique patients were included in the analyses, of whom 8 underwent a Blalock-Taussig shunt; 27, the Glenn procedure; 19, the Fontan procedure; 3, mechanical valve replacement; and 2, the Rastelli procedure. None of the patients with biological valve replacements remained in the study. The patients’ demographic details are shown in Table 1. The heparin infusion rates upon achieving the target APTTs for the 59 patients included in this study are shown in Fig. 2. The average time to reach target APTT was 25.5 h (range, 4.1–69.1 h). Of note, none of the patients developed heparin-induced thrombocytopenia, thrombosis or apparent bleeding before achieving target APTT after starting heparin therapy.

**Table 1:** Demographic details of the study patients.

Demographics	Number (n = 59)
Age (years)	0.8 (0–4)
Weight (kg)	7.5 (2.4–16.0)
Height (cm)	69.0 (41–105)
BMI	12.1–19.0 (15.1)
**Sex, n (%)**	
Male	38 (64.4)
Female	21 (35.6)
**Types of surgery, n (%)**	
Blalock-Taussig shunt	8 (13.6)
Glenn procedure	27 (45.8)
Fontan procedure	19 (32.2)
Biological valve replacement	0 (0.0)
Mechanical valve replacement	3 (5.1)
Rastelli procedure	2 (3.4)
**Cardiac diseases requiring surgery, n (%)**	
Pulmonary atresia	19 (32.2)
Hypoplastic left heart syndrome	10 (16.9)
Single ventricle	10 (16.9)
Tricuspid atresia	7 (11.9)
Double outlet right ventricle	6 (10.2)
Atrioventricular septal defect	2 (3.4)
Dilated cardiomyopathy	1 (1.7)
Complete transposition of great arteries	1 (1.7)
Prosthetic valve dysfunction	1 (1.7)
Mitral stenosis	1 (1.7)
Truncus arteriosus	1 (1.7)

*BMI* body mass index.

Values are expressed as either mean (range) or number (%).

**Figure 3:**
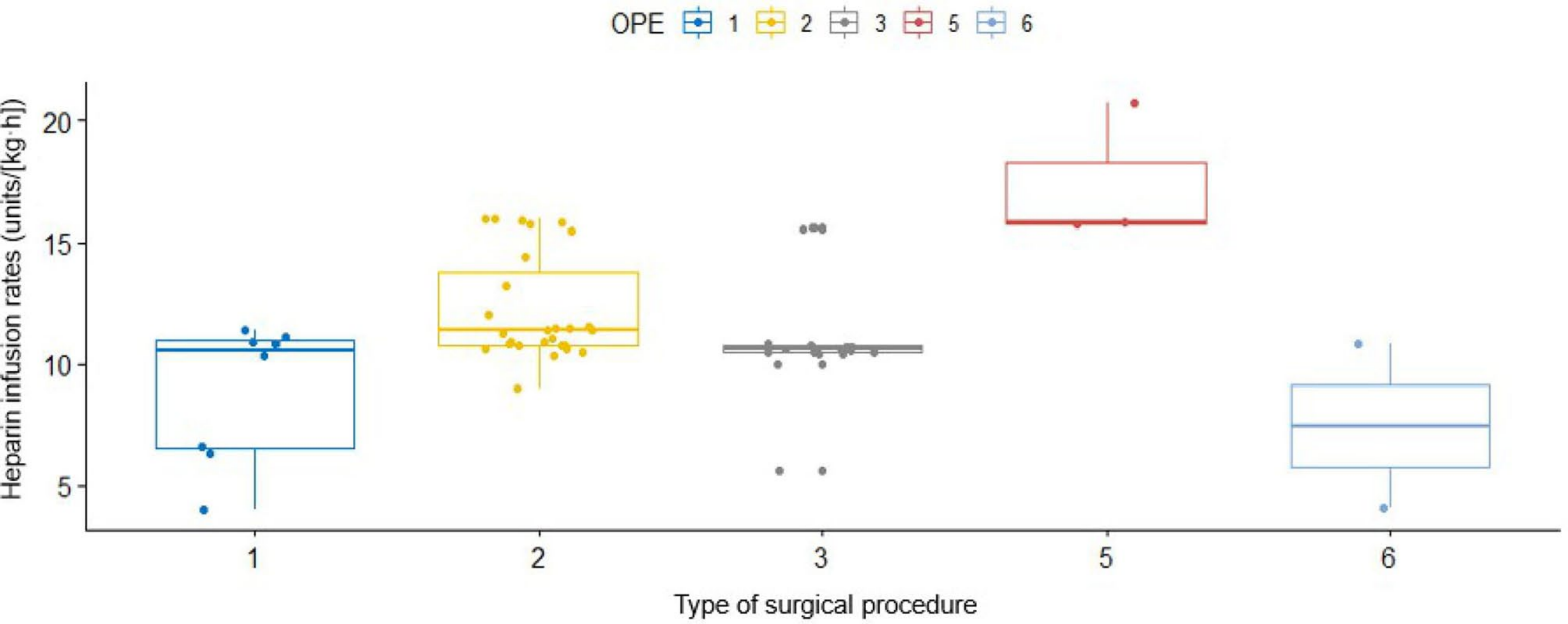
Infusion rates of heparin drip by type of surgical procedure. The distribution of each type of surgical procedure was as follows: 1, Blalock-Taussig shunt; 2, Glenn procedure; 3, Fontan procedure; 5, mechanical valve replacement; and 6, Rastelli procedure. The types of surgical procedure were significantly associated with the heparin infusion rates (P = 0.00073 by the Kruskal–Wallis test).

None of the 59 patients underwent measurement for factor VIII after starting the heparin treatment, and no platelet transfusions or protamine were administered within 48 h of achieving the target APTT after starting the heparin treatment; thus, no association analyses were performed on these aspects. Table 2 shows the results of the associations between each covariate and heparin infusion rate upon achieving the target APTTs. We additionally assessed the associations of antithrombin III level as a continuous variable, given that this variable is well known to be involved in heparin’s mechanism of action and was important in the pediatric ECMO study^19,20^, which resulted in finding no significant association (P = 0.32). The infusion rates of heparin drip by type of surgical procedure are shown in Fig. 3. The types of surgical procedure were significantly associated with the differences in heparin infusion rate (P = 0.00073; Fig. 3).

**Table 2:** Associations between the covariates and heparin infusion rates upon achieving target activated partial thromboplastin times.

Variable	*n* = 59	P Value
Age (years)	0.8 (0–4)	0.14
Weight (kg)	7.5 (2.4–16.0)	0.27
Male sex, n (%)	38 (64.4%)	0.41
**Type of surgery**^**a**^		
Blalock-Taussig shunt	8 (13.6%)	0.00073
Glenn procedure	27 (45.8%)	
Fontan procedure	19 (32.2%)	
Biological valve replacement	0 (0.0%)	
Mechanical valve replacement	3 (5.1%)	
Rastelli procedure	2 (3.4%)	
Fibrinogen (> 300 mg/dL)	26 (44.1%)	0.81
AT3 (< 60%)	14 (23.7%)	0.71
Platelet count (> 300,000/μL)	3 (5.1%)	0.24
Total protein (< 5.0 g/dL)^b^	37 (62.7%)	0.72
Albumin (< 3.0 g/dL)	15 (25.4%)	0.13
ALT > 100 IU/L	2 (3.4%)	0.69
Creatinine (> 0.8 mg/dL)	1 (1.7%)	0.54
Use of FFP (within 2 days before reaching the target APTT)	2 (3.4%)	0.41

In the middle column, the values are expressed as either mean (range) or number (%).

*AT3* antithrombin III, *ALT* alanine transaminase, *FFP* fresh frozen plasma, *APTT* activated partial thromboplastin time.

^a^For type of surgery, the P value was calculated using the Kruskal–Wallis test.

^b^Three patients had missing data.

**Figure 4:**
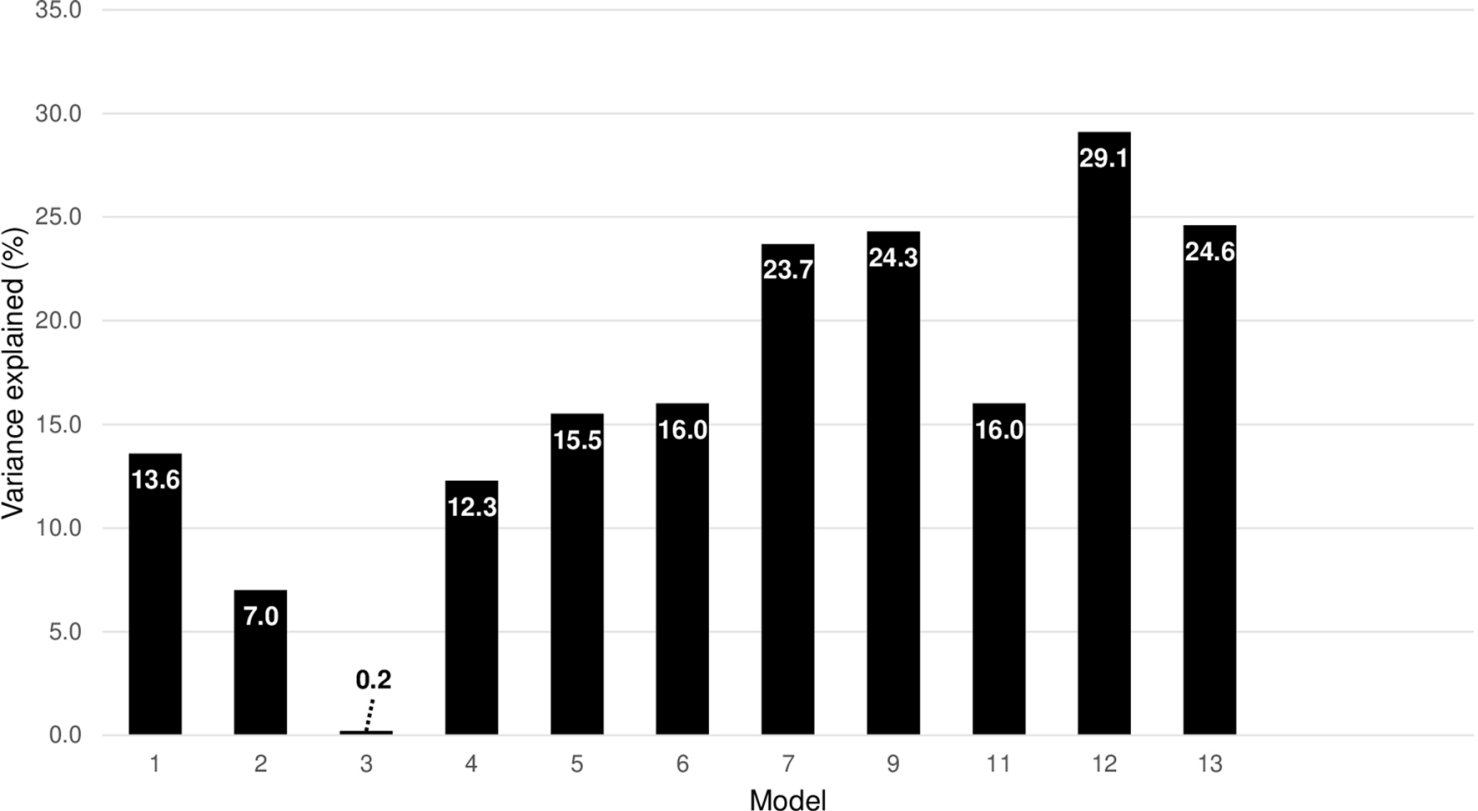
Variance explained by covariates associated with the interindividual variabilities of heparin infusion rate. We plotted the explained variance using the models. We constructed the models to account for each procedure as follows: model 1 for Blalock-Taussig shunt, model 2 for the Glenn procedure, model 3 for the Fontan procedure, model 4 for mechanical valve replacement, model 5 for Blalock-Taussig shunt plus Glenn procedure, model 6 for Blalock-Taussig shunt plus Fontan procedure, model 7 for Blalock-Taussig shunt plus mechanical valve replacement, model 9 for Glenn procedure plus mechanical valve replacement, model 11 for Blalock-Taussig shunt plus Glenn procedure plus Fontan procedure, model 12 for Blalock-Taussig shunt plus Glenn procedure plus mechanical valve replacement, and model 13 for Blalock-Taussig shunt plus Fontan procedure plus mechanical valve replacement.

Subsequently, the explained variance of these covariates was estimated by summing each explained variance. We constructed the models as shown in Supplemental Table 2. Each model considers the group of variables used for the multiple regression model. After rejecting the regression models where the directions of the regression coefficients are inconsistent with the analyses for each variable, only models 1–7, 9, and 11–13 remained, as shown in Table 3, Fig. 4, and Supplementary Table 3. The maximum variance explained among the models was from model 12, which was 29.1% (Fig. 4). The directions of the associations and AICs for each model are also shown in Table 3 and Supplementary Table 3. We also estimated the variance explained, adjusting for the APTT in each model, which resulted in no apparent changes in the results as compared with those without adjustment for the APTT (Supplementary Table 4).

**Table 3:** Coefficients of the models and percentages of explained variance in the dependent variables.

Component	Dependent variables of heparin infusion rate					
	Model 1	Model 2	Model 4	Model 7	Model 9	Model 12
Blalock-Taussig shunt	− 0.454***			− 0.418***		− 0.295*
Glenn procedure		0.224**			0.301***	0.220**
Fontan procedure						
Mechanical valve replacement			0.675***	0.615***	0.820***	0.738***
AIC value	63.1	67.5	64.0	57.8	57.3	55.4
Explained variance (%) CI (2.5%, 97.5%)	13.6 (0.46, 38.8)	7.0 (2.6, 20.3)	12.3 (0.0, 31.3)	23.7 (6.5, 49.7)	24.3 (10.3, 42.0)	29.1 (14.6, 52.5)

Only the models for which coefficients had P values of *< 0.1, **< 0.05, and ***< 0.01 are shown.

In total, 56 unique patients were included in the subgroup analyses, excluding three patients with mechanical valve replacement. We assessed the associations between covariates and heparin infusion rate. Age and type of surgical procedure were significantly associated with heparin infusion rate (P = 0.038 and 0.0044, respectively; Supplementary Table 5). Subsequently, the explained variance of the covariates was estimated. The constructed models are shown in Supplementary Table 6. Models 1–4, 7, 9, 11, and 12 remained after rejecting the regression models in which the directions of the regression coefficients are inconsistent. The maximum explained variance was from model 11, which was 27.5%, and the directions of the associations and AICs for each model are also shown in Supplementary Table 7. We also estimated the explained variance, adjusting for the APTT in each model, which resulted in no apparent changes in the results compared with those without adjustment for the APTT (models 1–4, 7–9, 11, and 12 remained after rejecting the regression models where the directions of the regression coefficients were inconsistent; Supplementary Table 8).

## Discussion

To the best of our knowledge, this is the first study to identify the extent of each attributing factors to the interindividual variability in unfractionated heparin therapy for patients not on ECMO who were admitted to the PICU after cardiac surgery. Our key finding is that over 70% of the interindividual variability in maintenance unfractionated heparin therapy was unexplained suggesting that responses to heparin therapy are difficult to predict. Factors/parameters from current practice which contributed to heparin dosing included the type of surgery in the main analysis, and age and the type of surgery in subgroup analysis. A similar study investigating variability in heparin dose response in the pediatric patients on ECMO was conducted using this approach by Moynihan et al. They showed that less than 50% of the variability was explained using a model incorporating age and antithrombin activity^19,20^. Other studies that assessed interindividual variability of other than heparin dose response were regarding capacity for motor recovery after ischemic stroke or sleeping metabolic rate. The results showed that the clinical variables explained 65–89% of the variance, which is obviously much higher than the rate in our study^21,22^. Thus, further investigation is needed to unveil the unknown factors contributing to the variabilities.

Unfractionated heparin interacts with antithrombin III and inhibits activated coagulation factors involved in clotting sequence^11,23,24^. Unfractionated heparin is bound to antithrombin, fibrinogens, globulins, serum proteases, and lipoproteins^11,23,25^. The anticoagulant effect is produced by inactivating thrombin and factor Xa^26^. It is mainly cleared by the liver and reticuloendothelial cells, and clearance starts by binding to proteins, endothelial cells, and macrophages^23^. It is also eliminated via the kidneys; however, some of the elimination pathways are still unknown^27,28^. Alternate explanations for variability in dosing could be explained by differences in pentasaccharide sequence content within the vials^29–31^ and the impact of developmental hemostasis^32–34^. As many of these factors are unmeasured in clinical practice, patient responses to unfractionated heparin therapy tend to be unpredictable.

In our study, we incorporated numerous factors from our clinical practice likely to contribute to heparin dose variability. We did not observe influences on heparin dosing from multiple factors that could affect the responses to heparin therapy as identified in previous studies^5–11^. The only known factors that showed any associations with the rate of heparin therapy upon achieving APTT were the type of surgery in the main analysis, and age and the type of surgery in subgroup analysis. Our inability to predict heparin dose requirements represents a clinical challenge.

Finally, we built a model with the maximum number of variables, resulting in the explained variance being < 30% (Table 3, Fig. 4, and Supplementary Table 3). In other words, when calculating the AIC for each model to assess the fitting into our data, even by the best model with the highest ability to predict, which was Model 12, accounting for Blalock-Taussig shunt plus the Glenn procedure and mechanical valve replacement, the variance explained was only 29.1%. We also ensured that other environmental factors related to the measurements of APTT did not change our results by adjusting for the APTT (Supplementary Table 3). Our results considering the type of surgery such as Blalock-Taussig shunt plus the Glenn procedure and mechanical valve replacement could contribute the most to the variabilities. Our finding that Blalock-Taussig shunt was negatively associated with the variabilities may bring new insights into the behavior of the variabilities after each surgery. A subsequent subgroup analysis was conducted to ensure that these findings were not biased by inclusion of patients with mechanical valve replacement for whom the target APTT was slightly higher than patients who underwent other surgical procedures. We then confirmed that the findings were very similar to the main analyses (Supplementary Tables 7 and 8), with the maximum explained variance being estimated by the best model, which accounted for type of surgical procedure having the lowest AIC.

Our findings suggest that type of surgery may explain some variability; however, we need to interpret this with caution. No previous studies have been conducted to investigate pathophysiological mechanisms which affect responses to heparin therapy between the type of cardiac surgeries. Furthermore, these associations are not modifiable. Further investigations are required to better explain our findings, improve heparin dose prediction and ultimately enhance patient care.

Obviously, many more factors should be taken into account to fully understand the interindividual variabilities, an example of which could be genetic factors. Genetic factors have proven to be important modulators of the metabolism of medications and can influence their efficacy and toxicity. Previous studies have reported that 20%–30% of the inter-individual differences in drug metabolism and drug response were estimated to be due to genetic variations^35,36^. Until now, no pharmacogenomic studies related to heparin therapy have been conducted, except for heparin-induced thrombocytopenia^37–39^. Including genetic factors could improve the prediction of interindividual variabilities and will be the next step to consider.

Our study has several limitations that must be acknowledged. First, the sample size was small, given the single-center study. Second, aPTT values differ between institutions and assay methods^7,40,41^. Third, the protocols of heparin therapy after cardiac surgery vary depending on the institution, and our aPPT targets and low heparin dose requirements present unique limiting generalizability^42–44^. Fourth, while we attempted to identify relevant covariates for our clinical practice, but this was limited by the retrospective nature of the study. Fifth, we only measured the first heparin dose required to achieve the therapeutic range, and changes over time were not evaluated. Finally, the influence of clinician bias in titrating heparin dose at the bedside according to surgery and other patient factors is not accounted for in our study design. Future studies should be conducted to explore this individual variation across other populations and evaluate associations with clinically relevant outcomes, such as bleeding and thrombosis.

This is the first study to show the variance explained by interindividual variabilities related to unfractionated heparin therapy in patients not on ECMO. Our study could lead to larger and well-designed prospective studies given that our findings seem to illuminate the need for further exploration, and ultimately these future studies might lead to the best possible post-cardiac heparin therapy for each patient.

## Conclusions

More than 70% of the interindividual variability in initial heparin maintenance dosing was unexplained. Further investigation of unknown factors is required to fully understand the interindividual variabilities, which could lead to the optimization of personalized heparin therapy for each patient.

## Supplementary information

Supplementary Information.

## Data Availability

The datasets used and/or analyzed during the current study are available from the corresponding author on reasonable request.

## Abbreviations

APTT: Activated partial thromboplastin time
ACT: Activated clotting time
ECMO: Extracorporeal membrane oxygenation
PK: Pharmacokinetics
PD: Pharmacodynamics
CPB: Cardiopulmonary bypass
PICU: Pediatric intensive care unit
NCCHD: National center for child health and development
AIC: Akaike information criterion
IQR: Interquartile range
BMI: Body mass index
AT3: Antithrombin III
ALT: Alanine transaminase
FFP: Fresh frozen plasma
CI: Confidence interval

## References

[1] Liveris A (2014). Anti-factor Xa assay is a superior correlate of heparin dose than activated partial thromboplastin time or activated clotting time in pediatric extracorporeal membrane oxygenation. Pediatr. Crit. Care Med..

[2] McLaughlin K (2019). Evaluation of antifactor-Xa heparin assay and activated partial thromboplastin time values in patients on therapeutic continuous infusion unfractionated heparin therapy. Clin. Appl. Thromb. Hemost..

[3] Saini S (2019). Anti-factor Xa-based monitoring of unfractionated heparin: clinical outcomes in a pediatric cohort. J. Pediatr..

[4] Wahking RA, Hargreaves RH, Lockwood SM, Haskell SK, Davis KW (2019). Comparing anti-factor Xa and activated partial thromboplastin levels for monitoring unfractionated heparin. Ann. Pharmacother..

[5] Olson JD (1998). College of American Pathologists Conference XXXI on laboratory monitoring of anticoagulant therapy: laboratory monitoring of unfractionated heparin therapy. Arch. Pathol. Lab. Med..

[6] Francis JL, Groce JB, Heparin Consensus G (2004). Challenges in variation and responsiveness of unfractionated heparin. Pharmacotherapy..

[7] Marlar RA, Clement B, Gausman J (2017). Activated partial thromboplastin time monitoring of unfractionated heparin terapy: issues and recommendations. Semin. Thromb. Hemost..

[8] Linhardt RJ, Claude S (2003). Hudson Award address in carbohydrate chemistry. Heparin. J Med Chem..

[9] Durrani J, Malik F, Ali N, Jafri SIM (2018). To be or not to be a case of heparin resistance. J. Community Hosp. Intern. Med. Perspect..

[10] Monagle P (2012). Antithrombotic therapy in neonates and children: antithrombotic therapy and prevention of thrombosis, 9th ed: American College of Chest Physicians Evidence-Based Clinical Practice Guidelines. Chest.

[11] Garcia DA, Baglin TP, Weitz JI, Samama MM (2012). Parenteral anticoagulants: antithrombotic therapy and prevention of thrombosis, 9th ed: American College of Chest Physicians Evidence-Based Clinical Practice Guidelines. Chest.

[12] Al-Sallami H, Newall F, Monagle P, Ignjatovic V, Cranswick N, Duffull S (2016). Development of a population pharmacokinetic-pharmacodynamic model of a single bolus dose of unfractionated heparin in paediatric patients. Br. J. Clin. Pharmacol..

[13] Delavenne X (2017). Pharmacokinetic/pharmacodynamic model for unfractionated heparin dosing during cardiopulmonary bypass. Br. J. Anaesth..

[14] Ito S (2011). Pharmacokinetics 101. Paediatr. Child Health..

[15] Ranucci M (2016). Fibrinogen levels after cardiac surgical procedures: Association with postoperative bleeding, trigger values, and target values. Ann Thorac Surg..

[16] Akaike H (1974). A new look at the statistical model identification. IEEE Trans. Autom. Control.

[17] Team RC. *R: A Language and Environment for Statistical Computing*. R Foundation for Statistical Computing, Vienna. https://www.R-project.org/ (2013).

[18] H W. *ggplot2: Elegant Graphics for Data Analysis*. Springer-Verlag New York. https://ggplot2.tidyverse.org (2016).

[19] Derbalah A, Duffull S, Newall F, Moynihan K, Al-Sallami H (2019). Revisiting the Pharmacology of Unfractionated Heparin. Clin. Pharmacokinet..

[20] Moynihan K (2017). Coagulation monitoring correlation with heparin dose in pediatric extracorporeal life support. Perfusion..

[21] Prabhakaran S (2008). Inter-individual variability in the capacity for motor recovery after ischemic stroke. Neurorehabil. Neural. Repair..

[22] Ganpule AA, Tanaka S, Ishikawa-Takata K, Tabata I (2007). Interindividual variability in sleeping metabolic rate in Japanese subjects. Eur. J. Clin. Nutr..

[23] Heparin sodium [package insert]. *U.S. Food and Drug Administration* https://www.accessdata.fda.gov/drugsatfda_docs/label/2017/017029s140lbl.pdf. (2017)

[24] Hirsh J, Anand SS, Halperin JL, Fuster V (2001). Mechanism of action and pharmacology of unfractionated heparin. Arterioscler. Thromb. Vasc. Biol..

[25] Smith GF, Sundboom JL (1981). Heparin and protease inhibition. I. Heparin complexes with thrombin, plasmin, and trypsin. Thromb. Res..

[26] Lam LH, Silbert JE, Rosenberg RD (1976). The separation of active and inactive forms of heparin. Biochem. Biophys. Res. Commun..

[27] Bara L, Billaud E, Gramond G, Kher A, Samama M (1985). Comparative pharmacokinetics of a low molecular weight heparin (PK 10 169) and unfractionated heparin after intravenous and subcutaneous administration. Thromb. Res..

[28] Bick RL (2002). Disorders of Thrombosis and Hemostasis: Clinical and Laboratory Practice.

[29] Alquwaizani M, Buckley L, Adams C, Fanikos J (2013). Anticoagulants: a review of the pharmacology, dosing, and complications. Curr. Emerg. Hosp. Med. Rep..

[30] De Caterina R (2007). Anticoagulants in heart disease: current status and perspectives. Eur. Heart J..

[31] Weitz DS, Weitz JI (2010). Update on heparin: what do we need to know?. J. Thromb. Thrombolysis..

[32] Andrew M, Vegh P, Johnston M, Bowker J, Ofosu F, Mitchell L (1992). Maturation of the hemostatic system during childhood. Blood.

[33] Monagle P (2006). Developmental haemostasis Impact for clinical haemostasis laboratories. Thromb. Haemost..

[34] Rahman M, George C, Monagle P (2020). Hot topics in coagulation testing: Important considerations for testing children for bleeding/thrombotic disorders. Int. J. Lab. Hematol..

[35] Lauschke VM, Ingelman-Sundberg M (2016). Requirements for comprehensive pharmacogenetic genotyping platforms. Pharmacogenomics..

[36] Sim SC, Kacevska M, Ingelman-Sundberg M (2013). Pharmacogenomics of drug-metabolizing enzymes: a recent update on clinical implications and endogenous effects. Pharmacogenom. J..

[37] Karnes JH (2018). Pharmacogenetics to prevent heparin-induced thrombocytopenia: what do we know?. Pharmacogenomics..

[38] Rollin J (2015). Increased risk of thrombosis in FcgammaRIIA 131RR patients with HIT due to defective control of platelet activation by plasma IgG2. Blood.

[39] Karnes JH (2015). A genome-wide association study of heparin-induced thrombocytopenia using an electronic medical record. Thromb Haemost..

[40] Greaves, M., & Control of Anticoagulation Subcommittee of the S, Standardization Committee of the International Society of T, Haemostasis. Limitations of the laboratory monitoring of heparin therapy. Scientific and Standardization Committee communications: On behalf of the Control of Anticoagulation Subcommittee of the Scientific and Standardization Committee of the International Society of Thrombosis and Haemostasis. *Thromb Haemost*. **87**, 163–164 (2002). 11848446

[41] Baluwala I, Favaloro EJ, Pasalic L (2017). Therapeutic monitoring of unfractionated heparin—trials and tribulations. Expert. Rev. Hematol..

[42] Service, N.H. *GG&C Paediatric Guidelines*. https://www.clinicalguidelines.scot.nhs.uk/ggc-paediatric-guidelines/ggc-guidelines/intensive-and-critical-care/cardiac-post-op-patients-anti-coagulation-therapy-in-picu/ (2020).

[43] Health U. *Therapeutic Dosing of Unfractionated Heparin: Neonatal/Pediatric Inpatient Clinical Practice Guideline*. https://www.uwhealth.org/files/uwhealth/docs/anticoagulation/Pediatric-Therapeutic-Dosing-Unfractionated-Heparin.pdf (2018).

[44] Health SCs. *Anticoagulation: Starship children’s health, Private Bag 92024, Auckland 1142, New Zealand*. https://www.starship.org.nz/guidelines/anticoagulation/ (2017).

