# Supplementary material for: Individual variation in unfractionated heparin dosing after pediatric cardiac surgery
Source: Sci Rep. 2020 Nov 10;10:19438. doi: 10.1038/s41598-020-76547-8 (PMC7655810; doi:10.1038/s41598-020-76547-8)
Supplement: Supplementary file 1 — Supplementary Information. [file 41598_2020_76547_MOESM1_ESM.docx]

**Individual variation in unfractionated heparin dosing after pediatric cardiac surgery**

**Keiko Hikino, M.D., Ph.D.^1,2^, Masaru Koido, Ph.D.^3^, Kentaro Ide, M.D.^1^, Nao Nishimura, M.D.^1^, Chikashi Terao, M.D., Ph.D.^3^, Taisei Mushiroda, Ph.D.^2^, Satoshi Nakagawa, M.D.^1^**

^1^Division of Critical Care Medicine, Department of Critical Care and Anesthesia, National Center for Child Health and Development, 2-10-1 Okura, Setagaya-ku, Tokyo 157-8535, Japan.

^2^Laboratory for Pharmacogenomics, RIKEN Center for Integrative Medical Sciences, 1-7-22 Suehiro-cho, Tsurumi-ku, Yokohama City, Kanagawa 230-0045, Japan.

^3^Laboratory for Statistical and Translational Genetics, RIKEN Center for Integrative Medical Sciences, 1-7-22 Suehiro-cho, Tsurumi-ku, Yokohama City, Kanagawa 230-0045, Japan.

**Correspondence:** Keiko Hikino, MD, PhD, Division of Critical Care Medicine, Department of Critical Care and Anesthesia, National Center for Child Health and Development, 2-10-1 Okura, Setagaya-ku, Tokyo 157-8535, Japan, and Laboratory for Pharmacogenomics, RIKEN Center for Integrative Medical Sciences, 1-7-22 Suehiro-cho, Tsurumi-ku, Yokohama City, Kanagawa 230-0045, Japan. E-mail: [keiko.hikino@riken.jp](mailto:keiko.hikino@riken.jp); Tel: +81-45-503-9598; Fax: +81-45-503-9568

Supplementary Table 1. Unfractionated heparin therapy for patients admitted at the pediatric intensive care unit of the National Center for Child Health and Development, Tokyo, Japan, after cardiac surgery

| **Name of cardiac surgery** | **APTT (seconds)** |
| --- | --- |
| Blalock-Taussig shunt | 40–60 |
| Glenn procedure | 40–60 |
| Fontan procedure | 40–60 |
| Biological valve replacement | 40–60 |
| Mechanical valve replacement | 45–65 |
| Rastelli procedure | 40–60 |

APTT, activated partial thromboplastin time

Supplementary Table 2. The variables used to construct the models.

| Model | Variable |
| --- | --- |
| 1 | Blalock-Taussig shunt |
| 2 | Glenn procedure |
| 3 | Fontan procedure |
| 4 | Mechanical valve replacement |
| 5 | Blalock-Taussig shunt plus Glenn procedure |
| 6 | Blalock-Taussig shunt plus Fontan procedure |
| 7 | Blalock-Taussig shunt plus mechanical valve replacement |
| 8 | Glenn procedure plus Fontan procedure |
| 9 | Glenn procedure plus mechanical valve replacement |
| 10 | Fontan procedure plus mechanical valve replacement |
| 11 | Blalock-Taussig shunt plus Glenn procedure plus Fontan procedure |
| 12 | Blalock-Taussig shunt plus Glenn procedure plus mechanical valve replacement |
| 13 | Blalock-Taussig shunt plus Fontan procedure plus mechanical valve replacement |
| 14 | Glenn procedure plus Fontan procedure plus mechanical valve replacement |
| 15 | All types of surgical procedures |

Supplementary Table 3. Coefficients of the models and percentages of explained variance in the dependent variables

|  | **Dependent variables of heparin infusion rate** | | | | | | | | | | |
| --- | --- | --- | --- | --- | --- | --- | --- | --- | --- | --- | --- |
| **Component** | **Model**  **1** | **Model**  **2** | **Model**  **3** | **Model**  **4** | **Model**  **5** | **Model**  **6** | **Model**  **7** | **Model**  **9** | **Model**  **11** | **Model**  **12** | **Model**  **13** |
| Blalock-Taussig shunt | −0.454*** |  |  |  | −0.387** | −0.509*** | −0.418*** |  | −0.481** | −0.295* | −0.455*** |
| Glenn procedure |  | 0.224** |  |  | 0.127 |  |  | 0.301*** | 0.033 | 0.220** |  |
| Fontan procedure |  |  | −0.045 |  |  | −0.147 |  |  | −0.119 |  | −0.093 |
| Mechanical valve replacement |  |  |  | 0.675*** |  |  | 0.615*** | 0.82*** |  | 0.738*** | 0.578** |
| **AIC value** | 63.1 | 67.5 | 71.6 | 64.0 | 63.8 | 63.4 | 57.8 | 57.3 | 65.4 | 55.4 | 59.0 |
| **Explained variance (%)**  **CI (2.5%, 97.5%)** | 13.6  (0.46, 38.8) | 7.0  (2.6, 20.3) | 0.2  (0.0, 9.3) | 12.3  (0.0, 31.3) | 15.5  (3.5, 40.4) | 16.0  (2.3, 42.5) | 23.7  (6.5, 49.7) | 24.3  (10.3, 42.0) | 16.0  (5.9, 50.8) | 29.1  (14.6, 52.5) | 24.6  (8.3, 52.8) |

AIC, Akaike information criterion; CI, confidence interval.

*P < 0.1; **P < 0.05; ***P < 0.01.

Supplementary Table 4. Coefficients of the models and percentages of explained variance in the dependent variables, adjusted for activated partial thromboplastin times

|  | **Dependent variables of heparin infusion rate** | | | | | | | | | |
| --- | --- | --- | --- | --- | --- | --- | --- | --- | --- | --- |
| **Component** | **Model**  **1** | **Model**  **2** | **Model**  **3** | **Model**  **4** | **Model**  **5** | **Model**  **6** | **Model**  **7** | **Model**  **9** | **Model**  **12** | **Model**  **13** |
| Blalock-Taussig shunt | −0.446*** |  |  |  | −0.390*** | −0.499*** | −0.401*** |  | −0.297* | −0.433*** |
| Glenn procedure |  | 0.210* |  |  | 0.110 |  |  | 0.278** | 0.195* |  |
| Fontan procedure |  |  | −0.039 |  |  | −0.140 |  |  |  | −0.077 |
| Mechanical valve replacement |  |  |  | 0.734*** |  |  | 0.668*** | 0.842*** | 0.761*** | 0.634*** |
| APTT | −0.007 | −0.004 | −0.008 | −0.012 | −0.005 | −0.006 | −0.010 | −0.007 | −0.007 | −0.010 |
| **AIC value** | 64.4 | 69.2 | 72.6 | 63.6 | 65.5 | 64.8 | 57.8 | 58.4 | 56.5 | 59.3 |
| **Explained variance (%)**  **CI (2.5%, 97.5%)** | 14.6  (3.5, 41.5) | 7.3  (0.97, 23.8) | 1.8  (0.15, 17.9) | 15.8  (3.0, 38.4) | 16.0  (5.5, 42.2) | 16.8  (7.0, 44.7) | 26.2  (12.3, 51.7) | 25.4  (13.0, 45.2) | 30.3  (18.0, 54.4) | 26.9  (14.8, 53.9) |

APTT, activated partial thromboplastin time; AIC, Akaike information criterion.

*P < 0.1; **P < 0.05; ***P < 0.01.

Supplementary Table 5. Associations between the covariates and heparin infusion rates upon achieving target activated partial thromboplastin times in the subgroup analyses.

| **Variable** | ***n* = 56** | **P Value** |
| --- | --- | --- |
| Age (years) | 0.8 (0–4) | **0.038** |
| Weight (kg) | 7.4 (2.4–16.0) | 0.19 |
| Male sex, n (%) | 35 (62.5%) | 0.71 |
| Type of surgery^a^  Blalock-Taussig shunt  Glenn procedure  Fontan procedure  Rastelli procedure | 8 (14.3%)  27 (48.2%)  19 (33.9%)  2 (3.6%) | **0.0044** |
| Fibrinogen (>300 mg/dl) | 25 (44.6%) | 0.67 |
| AT3 (<60%) | 13 (23.2%) | 0.55 |
| Platelet count (>300,000/μl) | 3 (5.4%) | 0.28 |
| Total protein (<5.0 g/dl)^b^ | 35 (62.5%) | 0.70 |
| Albumin (<3.0 g/dl) | 15 (26.8%) | 0.21 |
| ALT >100 IU/l | 1 (1.8%) | 0.29 |
| Creatinine (>0.8 mg/dl) | 1 (1.8%) | 0.46 |
| Use of FFP (within 2 days before reaching the target APTT) | 2 (3.6%) | 0.47 |

AT3, antithrombin 3; ALT, alanine transaminase; FFP, fresh frozen plasma; APTT, activated partial thromboplastin time.

^a^For type of surgery, the P value was calculated using the Kruskal-Wallis test.

^b^Three patients had missing data.

In the middle column, the values are expressed as either mean (range) or number (%).

Supplementary Table 6. The variables used to construct the models in the subgroup analysis.

| Model | Variable |
| --- | --- |
| 1 | Age |
| 2 | Blalock-Taussig shunt |
| 3 | Glenn procedure |
| 4 | Fontan procedure |
| 5 | Age plus Blalock-Taussig shunt plus Glenn procedure plus Fontan procedure |
| 6 | Age plus Blalock-Taussig shunt |
| 7 | Age plus Glenn procedure |
| 8 | Age plus Fontan procedure |
| 9 | Blalock-Taussig shunt plus Glenn procedure |
| 10 | Blalock-Taussig shunt plus Fontan procedure |
| 11 | Glenn procedure plus Fontan procedure |
| 12 | Age plus Blalock-Taussig shunt plus Glenn procedure |
| 13 | Age plus Blalock-Taussig shunt plus Fontan procedure |
| 14 | Age plus Glenn procedure plus Fontan procedure |
| 15 | Blalock-Taussig shunt plus Glenn procedure plus Fontan procedure |

Supplementary Table 7. Coefficients of the models and percentages of explained variance in the dependent variables in the subgroup analysis.

|  | **Dependent variables of heparin infusion rate** | | | | | | | |
| --- | --- | --- | --- | --- | --- | --- | --- | --- |
| **Component** | **Model**  **1** | **Model**  **2** | **Model**  **3** | **Model**  **4** | **Model**  **7** | **Model**  **9** | **Model**  **11** | **Model**  **12** |
| Age | 0.014 |  |  |  | 0.046 |  |  | 0.005 |
| Blalock-Taussig shunt |  | -0.418*** |  |  |  | -0.295*** |  | -0.290* |
| Glenn procedure |  |  | 0.301*** |  | 0.312*** | 0.220** | 0.584*** | 0.222* |
| Fontan procedure |  |  |  | 0.006 |  |  | 0.432*** |  |
| **AIC value** | 63.3 | 55.4 | 55.0 | 63.3 | 56.5 | 53.3 | 47.5 | 55.3 |
| **Explained variance (%)**  **CI (2.5%, 97.5%)** | 0.1  (0.0, 6.1) | 13.1  (0.18, 40.5) | 13.9  (3.7, 28.0) | 0.005  (0.0, 7.7) | 14.6  (4.6, 37.0) | 19.4  (7.1, 44.4) | 27.2  (9.6, 52.3) | 19.4  (8.0, 46.3) |

AIC, Akaike information criterion; CI, confidence interval.

*P < 0.1; **P < 0.05; ***P < 0.01.

Supplementary Table 8. Coefficients of the models and percentages of explained variance in the dependent variables, adjusted for activated partial thromboplastin times in the subgroup analysis.

|  |  | | **Dependent variables of heparin infusion rate** | | | | | | | |
| --- | --- | --- | --- | --- | --- | --- | --- | --- | --- | --- |
| **Component** | **Model**  **1** | **Model**  **2** | | **Model**  **3** | **Model**  **4** | **Model**  **7** | **Model**  **8** | **Model**  **9** | **Model**  **11** | **Model**  **12** |
| Age | 0.020 |  | |  |  | 0.047 | 0.018 |  |  | 0.006 |
| Blalock-Taussig shunt |  | -0.402*** | |  |  |  |  | -0.297* |  | -0.292* |
| Glenn procedure |  |  | | 0.279*** |  | 0.291*** |  | 0.197* | 0.567*** | 0.200* |
| Fontan procedure |  |  | |  | 0.019 |  | 0.008 |  | 0.423*** |  |
| APTT | -0.012 | -0.010 | | -0.006 | -0.012 | -0.007 | -0.012 | -0.007 | -0.003 | -0.007 |
| **AIC value** | 63.1 | 55.7 | | 56.3 | 63.2 | 57.7 | 65.1 | 54.5 | 49.3 | 56.5 |
| **Explained variance (%)**  **CI (2.5%, 97.5%)** | 3.8  (0.18, 23.5) | 15.8  (4.6, 42.4) | | 14.9  (5.3, 31.7) | 3.7  (0.2, 24.1) | 15.7  (6.6, 41.5) | 3.8  (0.47, 25.1) | 20.5  (10.0, 45.8) | 27.5  (11.5, 53.6) | 20.5  (11.7, 49.2) |

APTT, activated partial thromboplastin time; AIC, Akaike information criterion.

*P < 0.1; **P < 0.05; ***P < 0.01.
